# Consumption Patterns of Psychotropic Drugs Among Veterinary Medicine Students at the Federal University of Santa Maria

**DOI:** 10.3390/ijerph22121852

**Published:** 2025-12-11

**Authors:** Giovanne de Jesus Silva Pereira, Francini Arboit, Júlia Rosa Diniz, Eliane Maria Zanchet, Guilherme Vargas Bochi

**Affiliations:** 1 Center of Health Sciences, Department of Physiology and Pharmacology, Federal University of Santa Maria (UFSM), Santa Maria 97105-900, RS, Brazil; giovanne.pereira@acad.ufsm.br (G.d.J.S.P.); juliardiniz@gmail.com (J.R.D.); eliane.m.zanchet@ufsm.br (E.M.Z.); 2 Center of Rural Sciences, Postgraduate Program in Veterinary Medicine, Federal University of Santa Maria (UFSM), Santa Maria 97105-900, RS, Brazil; fraarboit@hotmail.com; 3 Center of Health Sciences, Postgraduate Program in Pharmacology, Federal University of Santa Maria (UFSM), Santa Maria 97105-900, RS, Brazil

**Keywords:** psychotropic drug use, mental health, academic stress, veterinary students

## Abstract

**Highlights:**

**Public health relevance—How does this work relate to a public health issue?**

- This study documents the high prevalence of psychotropic drug use among veterinary medicine students, reflecting substantial levels of anxiety, depression, and sleep disturbance in the young adult population.
- It focuses on future veterinary professionals, a group already known to be at increased risk for mental health problems and suicide, highlighting the need for early identification and support during training.

**Public health significance—Why is this work of significance to public health?**

- The study provides one of the first detailed profiles of psychotropic medication use among veterinary students in Brazil, identifying sex, sleep duration, and physical inactivity as key associated factors.
- By characterizing patterns of use, prescription sources, and treatment duration, the study generates evidence that can inform health promotion strategies within universities and health systems.

**Public health implications—What are the key implications or messages for practitioners, policy makers and/or researchers in public health?**

- Universities and health services should strengthen mental health support for veterinary students, including routine psychological screening, accessible counseling, and guidance on safe, supervised psychotropic use to reduce self-medication.
- Policymakers and practitioners can use these findings to design interventions that promote physical activity and sleep hygiene, aiming to reduce psychological distress and the reliance on pharmacological coping strategies in this population.

**Abstract:**

The rise in psychotropic drug use among students, particularly in Veterinary Medicine, correlates with high rates of mental disorders like depression and anxiety, often exacerbated by academic stress. Factors such as high academic demands, emotional exhaustion, and poor sleep quality contribute to the increased use of medications like antidepressants and anxiolytics. However, in Brazil, there is limited research on the profile and factors associated with this drug use among veterinary students. This study aims to assess the prevalence, patterns, and associated factors of psychotropic drug use at the Federal University of Santa Maria. A descriptive-correlational cross-sectional study was conducted using a survey covering sociodemographic data and psychotropic drug use. A total of 245 students participated in this study. The collected data included age, sex, semester, physical activity, sleep quality, drug use, family income, place of birth, and residence. In total, 36.7% of students reported using psychotropic medications during their undergraduate studies, with 77.1% being women and 22.9% men. Additionally, 45.6% reported insufficient sleep, defined as 4 to 6 h per day. Inactive students had a 94.5% higher likelihood of using psychotropic medications. Selective serotonin reuptake inhibitors (SSRIs) and benzodiazepines were the most reported drug classes. The findings highlight the emotional and academic burden of veterinary education, underscoring the need for institutional actions that prioritize student mental health.

## 1. Introduction

The use of psychotropic drugs is closely associated with mental disorders such as depression, anxiety, bipolar disorder, schizophrenia, and attention-deficit/hyperactivity disorder (ADHD). These conditions are among the leading causes of disability and significantly compromise the quality of life for both children and adults [1]. Studies consistently show that university students have higher rates of anxiety and depression than adults in the general population, likely due to academic and social stressors [2,3]. This heightened vulnerability is influenced by academic pressure, transitional life-stage challenges, and personal and financial stressors commonly experienced during higher education [4]. While high prevalence rates of mental distress are commonly observed across health sciences programs, with some studies reporting significant psychotropic drug use among medical students [5], this heightened vulnerability is influenced by academic pressure, transitional life-stage challenges, and personal and financial stressors commonly experienced during higher education. Some studies have examined the impact of academic demands on the mental health of students in health-related programs. For example, research at PUC-SP reported that 30.4% of medical students were using at least one psychotropic drug, mainly for anxiety and depression [5], and a study at the Federal University of Uberlândia found depressive-like symptoms in a large proportion of medical students [6]. Although these findings refer specifically to medical programs, they illustrate a similar pattern of psychological burden that may also affect students in other health fields, including Veterinary Medicine. Recent evidence also indicates that events such as the COVID-19 pandemic contributed to intensified anxiety, depression, and sleep disturbances among young adults, further increasing mental-health demands [7,8,9,10].

Veterinary Medicine students may face stressors related to the intense academic workload, ongoing study demands, and performance pressure. These aspects have been described as possible contributors to mental health problems such as anxiety and depression [11]. Studies also indicate that the combination of high cognitive demands, tight deadlines, and, for some students, exposure to emotionally challenging situations—such as animal suffering and euthanasia—can increase psychological vulnerability [12]. When present, these factors may contribute to elevated stress levels and negatively affect mental and physical health [13]. Concerns about the mental health of future veterinarians are further supported by evidence showing higher suicide rates in the profession compared to other healthcare fields [14]. Such factors may predispose students to coping strategies, including psychotropic drug use.

Despite growing attention to student mental health, few studies have specifically examined psychotropic drug use among Veterinary Medicine students in Brazil. Furthermore, little is known about treatment-seeking behavior, adherence, and the contextual factors associated with medication use in this population, representing a significant knowledge gap. Therefore, this study aimed to assess the prevalence, patterns, and associated factors of psychotropic drug use among Veterinary Medicine students at the Federal University of Santa Maria, providing evidence that may guide institutional mental-health strategies and support services.

## 2. Objective

- General Objective: To evaluate the use of psychotropic drugs among Veterinary Medicine students at the Federal University of Santa Maria (UFSM).
- Specific Objectives:
  - To determine the prevalence of psychotropic drug use among students;
  - To identify sociodemographic and behavioral factors associated with psychotropic drug use;
  - To characterize the classes of psychotropic medications used and their main clinical indications;
  - To assess patterns of treatment initiation and adherence, including prescription sources and duration of use.

## 3. Methodology

This is a descriptive correlational cross-sectional study conducted among Veterinary Medicine students at the Federal University of Santa Maria (UFSM), Brazil. The study aimed to assess the prevalence and associated factors of psychotropic drug use.

### 3.1. Participants

All undergraduate students enrolled in the Veterinary Medicine program at the Federal University of Santa Maria (UFSM), from the first to the tenth semester, were invited to participate voluntarily in the study. No randomization or specific selection process was applied. Of the total of 460 students enrolled in the undergraduate course, 245 responded to the questionnaires and were included in this study. The sample size was calculated using a 5% margin of error and a 95% confidence level, assuming significant heterogeneity within the population (50%). Based on these criteria and the total number of students enrolled in the Veterinary Medicine program for the first semester of 2024, it was determined that a representative sample should consist of at least 211 participants.

### 3.2. Questionnaire

Students from the first to the ninth semesters received the physical questionnaire. Tenth-semester students, who were off-campus for their final internship, completed the questionnaire electronically. The questionnaire comprised 23 questions. The first section of the questionnaire assessed sociodemographic factors, including gender, age, academic semester, living arrangements, geographic region, and physical activity engagement, among others. Then the second section focused on the use of psychotropic drugs, including whether the participant reported usage, duration of use, and the type of prescription. The questionnaire was developed by the authors based on instruments used in previous studies [5,15,16,17] investigating psychotropic drug use among university students. To ensure appropriateness of content and clarity, the questions were reviewed by faculty members with expertise in pharmacology and mental health. Although no pilot testing was performed, the instrument demonstrated adequate clarity during data collection.

### 3.3. Statistical Analysis

The sample size was calculated using the Epi Info™ software (StatCalc module version 7.2.5.0). Data collection was recorded in a Microsoft Excel spreadsheet, structured according to sociodemographic and psychotropic drug use variables. For statistical analysis, GraphPad Prism 9.0 (San Diego, CA, USA) and IBM SPSS Statistics 26 (IBM Corp., Armonk, NY, USA) software were used. A descriptive analysis of the variables was conducted to outline the profile of the students’ responses. Subsequently, Fisher’s Exact Test was applied to assess potential associations between categorical variables and psychotropic drug use, and the Unpaired Student’s *t*-test was used to compare continuous variables, such as mean age, between genders. In addition, binary logistic regression was used to evaluate the susceptibility of sex and engagement in physical activity to the use of psychotropic drugs during undergraduate studies. The selection of these two variables for the regression was based on their significance in the preliminary analyses, which provided the most robust interpretation of their effects. Statistical significance was set at *p* < 0.05.

### 3.4. Ethical Considerations

The study was approved by the Research Ethics Committee (CEP) of the Federal University of Santa Maria (UFSM), CAAE: 74080923.2.0000.5346. All participants voluntarily agreed to participate and provided written informed consent, authorizing the collection and use of their data in this research.

## 4. Results

### 4.1. Participant Profile

The study obtained responses from 52.5% of the veterinary course students, as indicated in Table 1. The analysis showed that the course is predominantly composed of women, accounting for 77.1% of the participants, with an average age of 21.9 years. Eighty-one percent of the students were originally from the southern region, followed by the southeastern region (13.5%). The family income ranged from 2 to 5 minimum wages per month for 48.6% of the participants, and the majority (39.6%) lived with family members. Additionally, among all participants, only 4.5% had attended the student support center (CAED), while 33.1% were unaware of its existence. Furthermore, as shown in Table 1, most participants (45.7%) reported sleeping fewer than the ideal number of hours, with only 36.7% of students sleeping between 7 and 8 h. Moreover, 55.9% of students engaged in regular physical activity. Finally, 53.9% of the students considered internships to be extracurricular activities.

### 4.2. Psychotropic Drug User Profile

**Figure 1:**
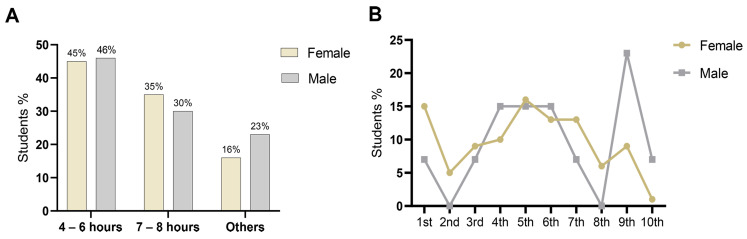
Patterns of psychotropic drug use among undergraduate students. (**A**) Percentage of students using psychotropic drugs according to average hours of sleep. (**B**) Percentage of students using psychotropic drugs according to semester of study.

Of the 245 participants, 36.7% (n = 90) reported using psychotropic drugs during their undergraduate studies, with a prevalence of 40.7% among women and 23.2% among men. However, most students, 63.3% (n = 155), stated that they had never used psychotropic drugs, as indicated in Table 2. Among the users, 81.1% were from the southern region, while 13.3% were from the southeastern region. Most users (51.1%; n = 46) had a family income ranging from 2 to 5 minimum wages per month. A significant portion of the participants who used psychotropic drugs lived alone (41.1%), although a notable number lived with family (37.8%). Moreover, most participants (53.3%) were engaged in internships as an extracurricular activity. Among the ten semesters of the course, the 1st and 5th semesters have the highest number of psychotropic drug users. A substantial part of the group that used these medications (45.6%) reported sleeping only 4 to 6 h per day (Figure 1A). Among female students, the first and fifth semesters showed the highest number of psychotropic drug users, with 15.6% and 16.9% (Figure 1B), respectively. In contrast, among male students, the fourth, sixth, and ninth semesters had the highest usage rates, at 15.4%, 15.4%, and 23.1%, respectively.

Most students reported initiating psychotropic drug use before entering the Veterinary Medicine program, as shown in Table 3, with approximately 67.8% already using such medications before enrollment. The number of psychotropic drugs consumed per individual ranged from one (reported by 37.8% of users) to four or more (13.3%). A substantial proportion of respondents (65.6%) had been using these medications for over 12 months. Regarding medical guidance, 72.2% indicated that the treatment was prescribed by a psychiatrist. Among all participants, 47.8% reported that the cost of psychiatric care was covered by health insurance. As shown in Table 3, the primary indication for psychotropic prescriptions was the treatment of anxiety, accounting for 34.4% of cases, although anxiety could be associated with other mental health disorders. Among the classes of psychotropic drugs used, Selective Serotonin Reuptake Inhibitors (SSRIs) were the most prevalent, with 26.6% of students undergoing treatment with these agents, followed by benzodiazepines (BDZs), reported by 13.5% of participants. Additionally, as shown in Table 4, binary logistic regression indicated a greater tendency toward psychotropic drug use among female students (Exp(β) = 2.27; *p* = 0.019). Regarding physical activity, students who were not physically active showed an estimated 94.5% increase in the odds of psychotropic drug use compared to active students (Exp(β) = 1.94; *p* = 0.013).

## 5. Discussion

The mental health of university students is increasingly discussed due to the impact of emotional distress on both the students and their future relationships with patients. Our results are consistent with others conducted in different regions of Brazil, showing a high prevalence of psychotropic drug use among students. However, while previous studies describe elevated rates among health-related majors, few investigations have focused explicitly on veterinary students, making our findings particularly relevant for an underexplored population. In the present study, 36.7% of veterinary students reported using psychotropic medications, with SSRIs being the most prevalent class, commonly used as treatment for anxiety and depressive disorders. This prevalence is comparable to, and in some instances higher than, what has been observed in other health-related fields. Studies with students from biological sciences, nursing, and medicine consistently report substantial psychotropic use during undergraduate training—whether due to anxiety, depressive symptoms, or academic pressure—indicating that veterinary students follow a broader pattern of psychological vulnerability already documented in health education programs [5,18,19,20,21,22,23].

Higher consumption of psychotropic medications among women has been consistently observed in Brazilian studies [19,20,21,24,25]. This pattern is associated with a greater frequency of mental disorder complaints among women, who are estimated to be about twice as likely as men to develop depression [26,27,28]. In our sample, the same pattern emerged, reinforcing sex-related vulnerability already identified in the literature but adding evidence specific to veterinary students, who are exposed to emotionally demanding training environments. In addition, women tend to identify signs and symptoms of illness more readily and are more proactive in seeking medical care [29]. Consequently, even when exposed to similar stressors, women show greater vulnerability to depressive and anxiety-related conditions, including post-traumatic stress disorder [30]. Depressive disorders commonly emerge in young adults, with peak incidence occurring between late adolescence and early adulthood [31]. Consistent with this pattern, the average age of psychotropic users in our sample was 21.9 years, particularly among women, which aligns with previous research indicating higher vulnerability in females aged 18–25 years [21,23,32,33].

Sleep disturbances also emerged as an essential component in this population. Although nearly half of the users reported adequate sleep duration, a substantial proportion experienced insufficient sleep, reinforcing the well-established association between sleep impairment, anxiety, and depressive symptoms [34,35,36]. Our findings suggest that sleep disturbances may both contribute to the need for psychotropic medication and persist despite treatment, indicating that these issues are not always resolved by pharmacotherapy alone. Insufficient sleep affects cognitive performance, emotional regulation, and coping capacity, contributing to psychological distress in university students, as previously demonstrated in Brazilian cohorts [37,38].

Overall, these findings highlight the intersection of developmental stage, sex-related vulnerability, and lifestyle factors—particularly sleep quality—as relevant contributors to psychotropic drug use among veterinary students. The integration of these variables in a single analysis represents one of the novel contributions of this study, enhancing understanding of how these factors converge in this academic population. In addition to the high rates of psychotropic use reported in the literature, an important protective factor observed in our study was the practice of regular physical activity. This protective association, while described in other student populations, has rarely been analyzed in veterinary students. Our findings, therefore, expand the evidence supporting exercise as a modifiable factor that institutions can promote to reduce psychological distress. This finding is consistent with Fasanella et al. (2022) [5] and reinforces the well-established role of exercise in mental health promotion. Physical activity is a recognized strategy for stress management and for reducing symptoms of depression and anxiety. Practices such as walking, running, yoga, and weight training can alleviate depressive symptoms, similar to cognitive-behavioral therapy, by increasing serotonin and dopamine levels and improving self-esteem and a sense of control [39]. Evidence shows that even 150 min per week of moderate-intensity exercise significantly lowers the risk of depression, as demonstrated in a meta-analysis with more than 2 million participants [40] and in studies with older adults with cancer [41]. Likewise, mind–body practices such as Tai Chi and Yoga produce moderate to strong effects [42,43].

In addition to physical aspects, the geographic origin of the students also appears to influence psychotropic drug use. Most students came from areas near the university, primarily from the same state. As reported by Vasconcelos et al. (2015) [44], living in the same region as the university and residing with family members are protective factors for mental health. This author also found that students from municipalities distant from the university, and thus away from their family environment, had a higher risk of developing depression. Furthermore, the South and Southeast regions of Brazil have the highest depression rates, at 15.2% and 11.5%, respectively. In contrast, the North and Northeast regions exhibit lower prevalence rates, with 4.1% in Pará and 4.2% in Amazonas [45].

The increased prevalence of depressive symptoms in the 1st and 5th semesters of the Veterinary Medicine program partially aligns with existing literature, especially regarding the beginning of university studies. Several studies suggest that starting higher education can be associated with high levels of stress, anxiety, and depression, due to a combination of personal expectations and social and academic pressures [46,47]. Our findings complement this evidence by identifying specific semesters that may require targeted institutional support within the veterinary curriculum. Regarding the 1st semester, our data agree with findings from Luna et al. (2017) [20], who, when analyzing psychotropic drug use among medical students, reported a prevalence of 23% among first-year students, with higher use among women (70%). These numbers suggest that the transition to higher education frequently involves excitement and frustration due to academic overload and limited practical experience [48].

Previous studies suggest that stress trajectories during veterinary and medical training may contribute to fluctuations in psychotropic drug use. Early in the program, students may rely on non-pharmacological coping strategies, but academic and emotional pressures tend to intensify in later semesters, especially due to clinical responsibilities and career uncertainty [49,50]. In addition, the demanding entrance process itself may predispose students to psychological distress, as prolonged pre-university study has been associated with early emotional exhaustion [48]. These factors align with our findings and reinforce that the mental health burden in health-related programs accumulates over time, highlighting the importance of early preventive support.

The predominance of Selective Serotonin Reuptake Inhibitors (SSRIs) among students is consistent with their role as first-line therapy for anxiety and depressive disorders due to their favorable efficacy and tolerability profiles [51,52]. This pattern reflects the psychological burden faced by veterinary students, who are frequently exposed to high academic demands and emotionally challenging situations during training [53]. In contrast, benzodiazepine use remains a concern given the risk of dependence and cognitive impairment [54]. Previous studies have shown that benzodiazepine misuse often stems from coping strategies for anxiety and sleep disturbances [55], which underscores the need for institutional mental-health programs that discourage unsupervised or symptomatic use. In this context, the use of psychotropic medications without specialized medical guidance represents a relevant concern. Although most students in our sample (72.2%) reported receiving psychiatric prescriptions, nearly 28% did not, suggesting that a portion may be engaging in self-medication as a strategy to cope with academic and emotional stressors. This practice may lead to inappropriate therapeutic management and increase the risk of adverse effects or dependence. Therefore, our findings highlight the need for improved institutional support and educational initiatives that promote safe, supervised access to mental health care among veterinary students.

Considering these findings, our study reinforces the importance of integrating preventive mental-health strategies into the veterinary curriculum, including routine psychological screening, accessible counseling services, structured referral pathways for psychiatric care, and educational modules on safe medication use and self-medication risks. Preventive measures such as physical activity programs, sleep hygiene campaigns, and peer support initiatives may help foster a healthier academic environment and reduce the need for medication-based coping strategies. Although this study provides important insights into psychotropic drug use among veterinary students, its novelty lies in consolidating multiple behavioral, demographic, and academic variables into a comprehensive mental-health profile of this population—something rarely documented in previous Brazilian studies. Nevertheless, future longitudinal and multicenter studies are warranted to explore causal pathways and regional differences in institutions.

This study has some limitations that should be acknowledged. First, the use of self-reported data may introduce recall bias and social desirability bias, as participants might have under- or over-reported information related to psychotropic drug use or mental health conditions. Second, although participation was voluntary, non-response bias may have occurred, particularly if students experiencing greater psychological distress chose not to participate. Additionally, the questionnaire was not piloted before data collection, which may have impacted the clarity of some questions and the accuracy of responses. Despite these limitations, the study provides relevant insight into psychotropic drug use patterns among veterinary students and highlights the need for institutional mental health support strategies.

## 6. Conclusions

This study identified a high prevalence of psychotropic medication use among veterinary students, with a greater likelihood among women and those reporting insufficient sleep and low physical activity. These findings reinforce the substantial emotional burden associated with veterinary training. Importantly, although most students received prescriptions from mental-health professionals, a considerable proportion reported unsupervised use, underscoring gaps in access to adequate psychological and psychiatric support. Collectively, the results highlight the need for universities to strengthen mental health services, promote preventive strategies such as sleep hygiene and physical activity, and implement educational initiatives on safe medication practices. Expanding institutional support systems may help reduce psychological distress and foster healthier academic environments for future veterinary professionals.

## Figures and Tables

**Table 1:** Participants’ General Characteristics.

Data	Total Sample	Female	Male
Matriculated = 467	$n\;(\%\;\mathrm{or}\;\bar{x}$ + SD)	$n\;(\%\;\mathrm{or}\;\bar{x}$ + SD)	$n\;(\%\;\mathrm{or}\;\bar{x}$ + SD)
**Participants**	245 (52.5%)	189 (77.1%)	56 (22.9%)
**Age**	21.9 ± 2.9	22.0 ± 3.0	23.3 ± 5.6
**Region of the Country**			
South	200 (81.6%)	159 (84.1%)	41 (73.2%)
Southeast	33 (13.5%)	22 (11.6%)	11 (19.6%)
Midwest	6 (2.4%)	4 (2.1%)	2 (3.6%)
Northeast	2 (0.8%)	2 (1.1%)	0 (0.0%)
North	3 (1.2%)	2 (1.1%)	1 (1.8%)
Other Countries	1 (0.5%)	0 (0.0%)	1 (1.8%)
**Family Income**			
Up to 1 minimum wage	10 (4.1%)	5 (2.6%)	5 (8.9%)
2–5 minimum wages	119 (48.6%)	95 (50.3%)	24 (42.9%)
6–10 minimum wages	80 (32.7%)	61 (32.3%)	19 (33.9%)
Over 10 minimum wages	34 (13.9%)	26 (13.8%)	8 (14.3%)
No response	2 (0.7%)	2 (1.0%)	0 (0.0%)
**Living Arrangements**			
Alone	82 (33.5%)	60 (31.7%)	22 (39.3%)
Family	97 (39.6%)	75 (39.7%)	22 (39.3%)
Friends	64 (26.1%)	52 (27.5%)	12 (21.4%)
No response	2 (0.8%)	2 (1.1%)	0 (0.0%)
**Physical Activity**			
Practice	137 (55.9%)	101 (53.4%)	36 (64.3%)
Do not practice	108 (44.1%)	88 (46.6%)	20 (35.7%)
**Extracurricular Activities**			
Internship	132 (53.9%)	104 (55.0%)	28 (50.0%)
Work	19 (7.8%)	13 (6.9%)	6 (10.7%)
Both	11 (4.4%)	6 (3.2%)	5 (8.9%)
No	83 (33.9%)	66 (34.9%)	17 (30.4%)
**Hours of Sleep**			
4–6 h	112 (45.7%)	85 (45.0%)	27 (48.2%)
7–8 h	90 (36.7%)	69 (36.5%)	21 (37.5%)
Others	38 (15.6%)	30 (15.9%)	8 (14.3%)
No response	5 (2.0%)	5 (2.6%)	0 (0.0%)
**Semester**			
1st semester	41 (16.7%)	31 (16.4%)	10 (17.9%)
2nd semester	23 (9.4%)	17 (9.0%)	6 (10.7%)
3rd semester	28 (11.4%)	23 (12.2%)	5 (8.9%)
4th semester	24 (9.8%)	19 (10.1%)	5 (8.9%)
5th semester	30 (12.2%)	23 (12.2%)	7 (12.5%)
6th semester	23 (9.4%)	19 (10.1%)	4 (7.1%)
7th semester	31 (12.7%)	23 (12.2%)	8 (14.3%)
8th semester	12 (4.9%)	11 (5.8%)	1 (1.8%)
9th semester	26 (10.6%)	20 (10.6%)	6 (10.7%)
10th semester	7 (2.9%)	3 (1.4%)	4 (7.1%)
CAED *			
Attend (ed)	11 (4.5%)	10 (5.3%)	1 (1.8%)
Never attended	116 (47.3%)	87 (46.0%)	29 (51.8%)
Does not know	81 (33.1%)	65 (34.4%)	16 (28.6%)
No response	37 (15.1%)	27 (14.3%)	10 (17.9%)

* CAED: Stands for “Support Center for Students”.

**Table 2:** Characteristics of Psychotropic Drug Users.

Data	Total of Participants	Female	Male	Fisher’s Exact Test
	$n\;(\%\;\mathrm{or}\;\bar{x}$ + SD)	$n\;(\%\;\mathrm{or}\;\bar{x}$ + SD)	$n\;(\%\;\mathrm{or}\;\bar{x}$ + SD)	*p* < 0.05
**Users**	90 (36.7%)	77 (40.7%)	13 (23.2%)	0.0181 *
**Non-users**	155 (63.3%)	112 (59.3%)	43 (76.8%)	
**Age**	21.9 ± 2.9	22.6 ± 3.4	26.6 ± 9.6	ns **
**Region of the Country**				
South	73 (81.1%)	64 (83.1%)	9 (69.2%)	ns
Southeast	12 (13.3%)	11 (14.3%)	1 (7.7%)	
Midwest	3 (3.3%)	1 (1.3%)	2 (15.4%)	
Northeast	1 (1.1%)	1 (1.3%)	0 (0.0)	
North	0 (0.0)	0 (0.0)	0 (0.0)	
Other Countries	1 (1.1%)	0 (0.0)	1 (7.7%)	
**Family Income**				
Up to 1 minimum wage	3 (3.3%)	3 (3.9%)	0 (0.0%)	ns
2–5 minimum wages	46 (51.1%)	39 (50.6%)	7 (53.8%)	
6–10 minimum wages	24 (26.7%)	22 (28.6%)	2 (15.4%)	
Over 10 minimum wages	17 (18.9%)	13 (16.9%)	4 (30.8%)	
**Living Arrangements**				
Alone	37 (41.1%)	30 (39.0%)	7 (53.8%)	ns
Family	34 (37.8%)	29 (37.7%)	5 (38.5%)	
Friends	18 (20.0%)	17 (22.1%)	1 (7.7%)	
No response	1 (1.1%)	1 (1.2%)	0 (0.0%)	
**Physical Activity**				
Yes	41 (45.6%)	34 (44.2%)	7 (53.8%)	ns
No	49 (54.4%)	43 (55.8%)	6 (46.2%)	
**Extracurricular Activity**				
Internship	48 (53.3%)	40 (51.9%)	8 (61.5%)	ns
Work	7 (7.8%)	5 (6.5%)	2 (15.4%)	
Both	6 (6.7%)	5 (6.5%)	1 (7.7%)	
No	29 (32.2%)	27 (35.1%)	2 (15.4%)	
**Hours of Sleep**				
4–6 h	41 (45.6%)	35 (45.5%)	6 (46.2%)	ns
7–8 h	31 (34.4%)	27 (35.1%)	4 (30.8%)	
Others	16 (17.8)	13 (16.9%)	3 (23.1%)	
No response	2 (2.2%)	2 (2.6%)	0 (0.0%)	
**Semester**				
1st semester	13 (14.4%)	12 (15.6%)	1 (7.7%)	ns
2nd semester	4 (4.4%)	4 (5.2%)	0 (0.0%)	
3rd semester	8 (8.9%)	7 (9.1%)	1 (7.7%)	
4th semester	10 (11.1%)	8 (10.4%)	2 (15.4%)	
5th semester	15 (16.7%)	13 (16.9%)	2 (15.4%)	
6th semester	12 (13.3%)	10 (13.0%)	2 (15.4%)	
7th semester	11 (12.2%)	10 (13.0%)	1 (7.7%)	
8th semester	5 (5.6%)	5 (6.5%)	0 (0.0%)	
9th semester	10 (11.1%)	7 (9.1%)	3 (23.1%)	
10th semester	2 (2.2%)	1 (1.3%)	1 (7.7%)	

** Unpaired *t*-test; * Significance; ns: no significance.

**Table 3:** Characteristics of the Psychotropic Drugs.

Data	Total Sample	Female	Male	Fisher’s Exact Test
	*n* (%)	*n* (%)	*n* (%)	*p* < 0.05
**Users**	90 (36.7%)	77 (40.7%)	13 (23.2%)	0.0181 *
**Onset of Use**				
Pre-college	61 (67.8%)	55 (71.4%)	6 (46.2%)	ns
In college	29 (32.2%)	22 (28.6%)	7 (53.8%)	
**Quantity of Psychotropic Drugs**				
1	34 (37.8%)	26 (33.8%)	8 (61.5%)	ns
2	26 (28.9%)	24 (31.2%)	2 (15.4%)	
3	18 (20.0%)	16 (20.8%)	2 (15.4%)	
≥4	12 (13.3%)	11 (14.3%)	1 (7.7%)	
**Time of Use**				
1–3 months	13 (14.4%)	10 (13.0%)	3 (23.1%)	ns
4–6 months	6 (6.7%)	4 (5.2%)	2 (15.4%)	
7–12 months	12 (13.3%)	12 (15.6%)	0 (0.0%)	
Over 12 months	59 (65.6%)	51 (66.2%)	8 (61.5%)	
**Psychiatric Prescription**				
Yes	65 (72.2%)	59 (75.6%)	6 (50%)	ns
Another specialty	15 (16.7%)	12 (15.4%)	3 (25%)	
Self-prescribed	9 (10.0%)	6 (7.7%)	3 (25%)	
No response	1 (1.1%)	1 (1.3%)	0 (0.0%)	
**Type of Prescription**				
SUS	9 (10%)	8 (10%)	1 (10%)	ns
Private	37 (41.1%)	32 (40%)	5 (50%)	
Heath Insurance	43 (47.8%)	40 (50%)	3 (30%)	
Other type	1 (1.1%)	0 (0.0%)	1 (10%)	
**Clinical Diagnoses**				
Anxiety	72 (34.4%)	65 (34.8%)	7 (31.0%)	ns
Depression	57 (27.3%)	52 (27.8%)	5 (22.7%)	
Insomnia	29 (13.9%)	23 (12.3%)	6 (27.3%)	
ADHD	24 (11.5%)	21 (11.2%)	3 (13.6%)	
Bipolar	11 (5.3%)	11 (5.9%)	0 (0.0%)	
Others	14 (6.7%)	13 (7.0%)	1 (4.5%)	
No response	2 (1.0%)	2 (1.1%)	0 (0.0%)	
**Class**				
Tricyclic	5 (2.4%)	5 (2.7%)	0 (0.0%)	ns
Tetracyclic	6 (2.9%)	6 (3.3%)	0 (0.0%)	
SSRIs	55 (26.6%)	52 (28.3%)	3 (13%)	
SNRIs	15 (7.2%)	13 (7.1%)	2 (8.7%)	
Atypical	13 (6.3%)	12 (6.5%)	1 (4.3%)	
Benzodiazepines	28 (13.5%)	26 (14.1%)	2 (8.7%)	
Buspirone	3 (1.4%)	2 (1.1%)	1 (4.3%)	
Psychostimulants	18 (8.7%)	15 (8.2%)	3 (13%)	
Hypnotics	30 (14.5%)	24 (13%)	6 (26.1%)	
Antipsychotics	6 (2.9%)	5 (2.7%)	1 (4.3%)	
Mood Stabilizers	9 (4.3%)	8 (4.3%)	1 (4.3%)	
Antiepileptic Drugs	18 (8.7%)	15 (8.2%)	3 (13.0%)	
No response	1 (0.5%)	1 (0.5%)	0 (0.0%)	

* Significance; ns: no significance.

**Table 4:** Simple Binary Logistic Regression to evaluate the impact of sex and physical activity on the dependent variable (psychotropic drug use).

Dependent Variable	Predictor	Β	Seβ	Wald	df	*p*	Exp(β)	Exp(β) (Lower 95% CI)
**Psychotropics** **Drugs Use**	**Female**	0.822	0.349	5.528	1	0.019 *	2.274	1.146
	Intercept	−1.196	0.317	14.285	1	0.000	0.302	
**Psychotropics** **Drugs Use**	**Physical** **Inactivity**	0.665	0.269	6.129	1	0.013 *	1.945	1.149
	Intercept	−0.851	0.187	20.795	1	0.000	0.427	

β: coefficient; Seβ: coefficient standard error; Wald: test statistics for the variables in the equation; df: degrees of freedom; Exp(β): Odds Ratio; CI: confidence interval; * Significant, within 95% confidence interval.

## Data Availability

The original contributions presented in this study are included in the article. Further inquiries can be directed to the corresponding author.
